# No dose adjustment of tigecycline is necessary during continuous renal replacement therapy: we are not sure

**DOI:** 10.1186/s13054-020-2775-0

**Published:** 2020-02-20

**Authors:** Patrick M. Honore, Cristina David, Luc Kugener, Sebastien Redant, Rachid Attou, Andrea Gallerani, David De Bels

**Affiliations:** ICU Department, Centre Hospitalier Universitaire Brugmann, Place Van Gehuchtenplein, 4, 1020 Brussels, Belgium

Broeker et al. conclude that tigecycline (TGC) losses during continuous renal replacement therapy (CRRT) are comparable to those of patients with normal renal function, indicating that no dose adjustment of TGC is necessary during CRRT [1]. We would like to make some comments. First, the RRT modality chosen may have influenced TGC elimination. TGC clearance during continuous veno-venous hemodiafiltration (CVVHDF) was more efficient (2.71 L/h) as compared to continuous veno-venous hemodialysis (CVVHD) (1.69 L/h) [1]. Second, Broeker et al. attribute the increased clearance of TGC with CVVHDF to low plasma protein binding (*f*u recently reported as 50–70%, compared to the previously reported 11–29%) [1], allowing better elimination through ultrafiltration [1]. This increased ultrafiltration yields a saturation coefficient of 0.79 for CVVHD and 0.90 for CVVHDF and probably higher for CVVH [1]. The removal by CVVHDF and CVVHD yield together a value of 11.2% [1]. If we look at CVVHD alone (1.69 L/h), this represents only 9% of the total body clearance (18.3 L/h) [1]. Looking at CVVHDF (2.71 L/h), this represents almost 15%. Third, TGC protein binding is affected by divalent cations such as calcium, and accordingly, regional citrate anticoagulation (RCA) might affect membrane transfer [1]. This would be suspected if convection was used in the study as it is more protein binding dependent. RCA was only used in CVVHD and not in CVVHDF, where unfractionated heparin (UHF) was used [1, 2]. Fourth, TGC can be adsorbed by plastic labware [3] and there is a great suspicion that TGC could be adsorbed by highly adsorptive membranes (HAM) [4]. Broeker et al. used a polysulfone membrane which is poorly adsorptive [3]; nevertheless, in their study, they observed a time delay in the effluent concentrations in one patient that may have been caused by adsorption losses inside the membrane [1]. They concluded that since the delay indicated a saturable binding, adsorption losses did not impact the dialysis clearance significantly [1]. We respectfully disagree, as most of the adsorption of small molecules does not occur at the surface of the membrane but rather occurs inside the membrane fibers and therefore it takes more time to become saturated [5]. Indeed, Tian et al. clearly demonstrated that the absence of saturation could exclude surface adsorption, as repeated doses of amikacin resulted in further bulk adsorption [5]. Overall, the conclusion that no dose adjustment is necessary during CRRT seems somewhat premature.

## Authors’ response

A. Broeker, C. Dorn, A. Kratzer, F. Kees, A. Heininger, M.G. Kees, H. Häberle, S.G. Wicha

We appreciate the comments by Honore et al. on our article [1]. We agree that with the data at hand, we cannot extrapolate to other RRT settings and membrane types than the ones investigated in our study. In fact, these points were stated by us as limitation of the study [1].

It is correct that RRT clearance was estimated to approx. 11.2% of the body clearance of tigecycline [1]. The mass balance analysis was presented including 10th–90th percentiles to indicate the variability in this vulnerable population. This in mind, we want to emphasize that even the 90th percentile evaluated (18.3% of the total elimination via CRRT) in patients suffering from acute kidney injury would not differ in a clinically relevant extent from the renal clearance (13% of the total elimination via renal elimination (SPC Tygacil 50 mg powder for solution for infusion, Pfizer Limited, Sandwich, UK) of non-renally impaired subjects).

Our study investigated two different RRT types (CVVHD and CVVHDF), but we were cautious to draw strong conclusions about differences between these two modes, given the modest differences observed and the small sample size. The speculation on the role of the anticoagulant used and its effect on protein binding is certainly a perspective for future research.

We agree with Honore et al. that adsorption is an underappreciated phenomenon in RRT clearance and requests deeper investigation as also stated in the limitations [1]. In vitro adsorption of tigecycline to polysulphone membranes was investigated by Sevillano et al. [6] where no issues regarding adsorptive loss were found in protein-containing medium (Mueller-Hinton broth) supporting our findings. Moreover, in our study [1], the post-filter plasma concentrations were collected but not used in the original analysis. In the patient where a delay was observed, a stable saturation coefficient ((*c*_pre-filter_ − *c*_post-filter_)/c_pre-filter_) over time (*t*_1h_ = 0.520, *t*_6h_ = 0.461, *t*_12h_ = 0.617) indicated minor non-linearity due to possible adsorption processes. Nonetheless, it seems likely that adsorptive loss was not having a relevant impact on the investigated population but more data, for instance with other membrane material as laid out in the limitations, and more sophisticated methods to determine adsorption in RRT are desirable.

To summarize, we disagree with Honore et al. that a different conclusion should be drawn from our study, but agree that more research is necessary to inform about dosing of tigecycline in other, not yet investigated RRT settings.

## Data Availability

Not applicable.

## Abbreviations

CRRT: Continuous renal replacement therapy
TGC: Tigecycline
CVVHDF: Continuous veno-venous hemodiafiltration
CVVHD: Continuous veno-venous hemodialysis
CVVH: Continuous veno-venous hemofiltration
IHD: Intermittent hemodialysis
RCA: Regional citrate anticoagulation
UFH: Unfractionated heparin
HAM: Highly adsorptive membranes
